# NLRP3 inflammasome activation is associated with type 1 inflammation and neutrophil activation in pyoderma gangrenosum across human and murine models

**DOI:** 10.1016/j.xjidi.2026.100512

**Published:** 2026-07-31

**Authors:** Takeshi Araki, Akihiko Uchiyama, Rimu Takata, Keiji Kosaka, Mayu Ohtaka, Shintaro Saito, Takayuki Shuto, Akiko Sekiguchi, Sachiko Ogino, Yoko Yokoyama, Ryoko Torii, Yuki Watanuki, Sei-ichiro Motegi

**Affiliations:** 1 Department of Dermatology, Gunma University Graduate School of Medicine, Maebashi, Japan

**Keywords:** Brequinar, Mouse model, Neutrophil extracellular traps, Transcriptional analysis, Type 1 inflammation

## Abstract

Pyoderma gangrenosum (PG) is a rare neutrophilic dermatosis characterized by painful, nonhealing cutaneous ulcers. Although dysregulated innate immunity and neutrophil activation are implicated in its pathogenesis, the underlying molecular mechanisms remain poorly defined. This study aimed to identify conserved pathogenic mechanisms by integrating analyses of human PG lesions and a brequinar-induced PG-like murine model and to assess therapeutic relevance. Skin biopsy specimens from patients with PG and publicly available RNA-sequencing data were analyzed alongside the murine model using histological and transcriptomic approaches. Cross-species transcriptomic integration identified shared inflammatory pathways between human and murine PG. Human PG lesions showed increased neutrophil infiltration, neutrophil extracellular trap formation, and a dominant type 1 inflammatory profile, all of which were recapitulated in the brequinar-induced mouse model, together with impaired wound healing. Transcriptomic analyses revealed enhanced oxidative stress responses and activation of the NLRP3/caspase-1 inflammasome pathway as a conserved pathogenic axis. In the murine model, both prednisolone and avacopan suppressed inflammatory cytokine expression and neutrophil activation; however, only avacopan significantly improved wound healing. Collectively, these findings suggest that NLRP3 inflammasome activation is associated with type 1 inflammation and neutrophil extracellular trap formation and may represent a PG-associated inflammatory pathway.

## Introduction

Pyoderma gangrenosum (PG) is a rare and severe neutrophilic dermatosis characterized by extreme pain, rapidly progressive ulceration, and frequently refractory clinical courses. Approximately half of the patients with PG have associated systemic disorders, such as inflammatory bowel disease, monoclonal gammopathy, hematologic malignancy or paraproteinemia, Sweet’s syndrome, hepatitis, HIV infection, systemic lupus erythematosus, pregnancy, and Takayasu arteritis (Binus et al, 2011). These associations support the concept that dysregulated innate immunity in a host predisposed to exaggerated neutrophil activation plays a central role in disease development. Accumulating evidence indicates that type 1 inflammatory responses, neutrophil extracellular trap (NET) formation, and aberrant cytokine production contribute to PG pathogenesis (Eid et al, 2021; Ogawa et al, 2021). NETs are now recognized not only for their antimicrobial function but also for their role in sterile inflammation and disease pathogenesis, with ROS production constituting a key step in NETosis (Papayannopoulos, 2018). Notably, complement component C5a has recently emerged as a critical mediator linking complement activation to neutrophil recruitment and NET formation (Wang et al, 2024). Despite these advances, mechanistic studies of PG have been limited by the rarity of the disease and the risk of pathergy, which can exacerbate ulceration after biopsy and restrict the availability of well-characterized human tissue samples. Current evidence-based treatments for PG include systemic corticosteroids, cyclosporine, and TNF-α inhibitors such as infliximab and adalimumab, which carry the highest levels of clinical recommendation (Yamamoto et al, 2023). More recently, case reports have described clinical responses to Jak inhibitors and biologics targeting IL-17 or IL-23 (Flora et al, 2025; Rijal et al, 2024; Wanzenberg et al, 2025). In parallel, clinical trials evaluating vilobelimab, a mAb targeting C5a, have been conducted in patients with PG (Lu et al, 2020). Nevertheless, many patients continue to experience persistent or recurrent disease despite combination immunosuppressive therapies, underscoring an unmet need to better define the innate immune pathways driving PG and to identify novel therapeutic targets.

A major impediment to progress in PG research has been the historical lack of a reproducible in vivo model that faithfully recapitulates the inflammatory and ulcerative features of the disease. In 2023, a novel PG-like mouse model involving topical administration of pyrimidine synthesis inhibitors, including N-phosphonacetyl-L-aspartate and brequinar (dihydroorotate dehydrogenase), was reported (Jatana et al, 2023). The application of N-phosphonacetyl-L-aspartate induced chronic nonhealing ulcers accompanied by robust NET formation and enhanced type 1 and type 3 inflammatory responses, closely mimicking key aspects of human PG. Although brequinar was also shown to induce PG-like lesions with NET formation in this study, the underlying molecular and immunological mechanisms remained incompletely characterized.

In this study, we validated and optimized the brequinar-induced PG mouse model and performed integrative transcriptomic analyses using publicly available human PG RNA-sequencing data together with newly generated transcriptomic datasets from murine PG-like skin lesions. Our results demonstrate that activation of the NLRP3/caspase-1 inflammasome is associated with enhanced type 1 inflammation, characterized by increased expression of IFN-γ–associated chemokines and neutrophil recruitment with NET formation, which is associated with inflammatory responses observed in both human PG lesions and the murine PG-like model. Furthermore, we show that pharmacological inhibition of C5a signaling with avacopan, an oral C5a receptor antagonist, ameliorates disease severity in the brequinar-induced PG mouse model, highlighting the C5a pathway as a potential therapeutic target in PG.

## Results

### Increased neutrophil infiltration and NET formation in lesional skin from patients with PG

**Figure 1:**
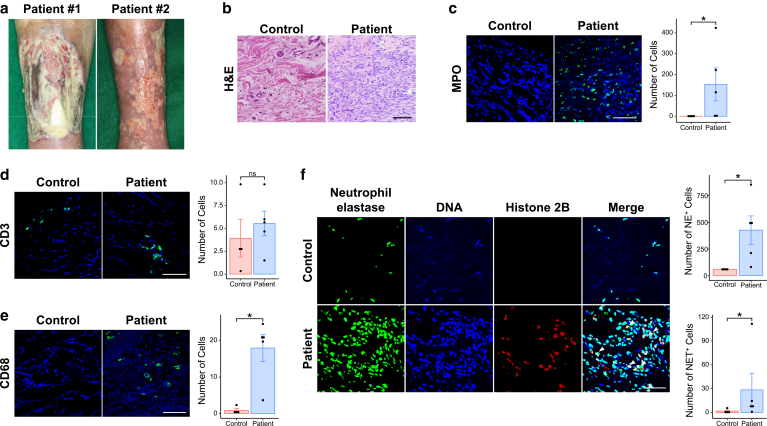
**Increased neutrophil infiltration and NET formation in lesional skin from patients with PG.** (**a**) Representative clinical features of patients with PG. (**b**) Representative H&E staining showing increased inflammatory cell infiltration in lesional skin from patients with PG compared with that in healthy control skin. (**c–e**) Representative immunofluorescence images showing (**c**) MPO^+^ neutrophils, (**d**) CD3^+^ T cells, and (**e**) CD68^+^ macrophages in healthy control skin and PG lesional skin. Quantification of positive cells is shown in the right panels and was performed by counting cells in 6 randomly selected fields per section. n = 5 per group. Bar = 50 μm. (**f**) Representative immunofluorescence images of healthy control skin and PG lesional skin showing neutrophil elastase–positive cells and NET formation. Neutrophil elastase (green), DNA (blue), and histone H2B (red) are shown. White arrows indicate areas positive for neutrophil elastase and histone H2B, consistent with NET formation. Bar = 50 μm. n = 4 for control and n = 5 for PG. Data are expressed as mean ± SEM. ∗*P* < .05. MPO, myeloperoxidase; NET, neutrophil extracellular trap; PG, pyoderma gangrenosum.

To characterize the inflammatory cell composition in human PG, we analyzed skin biopsy specimens from patients with PG (Figure 1a) and compared them with normal human skin controls. H&E staining demonstrated marked inflammatory cell infiltration extending from the ulcer margin into the surrounding dermis in PG lesions **(**Figure 1b). Immunofluorescence analysis revealed a significant increase in myeloperoxidase (MPO)^+^ neutrophils in PG lesions compared with those in normal skin (Figure 1c). In contrast, the number of CD3^+^ T cells did not differ significantly between PG lesions and normal skin (Figure 1d). CD68^+^ macrophages were significantly increased in PG lesions relative to that in the controls (Figure 1e). Furthermore, double immunofluorescence staining for neutrophil elastase (NE) and histone H2B revealed a pronounced increase in NE^+^ cells and NET formation in PG lesions compared with that in normal skin (Figure 1f). These findings indicate that excessive neutrophil activation and NET formation are characteristic features of lesional skin in patients with PG.

### Enhanced type 1 inflammatory responses in lesional skin in patients with PG

**Figure 2:**
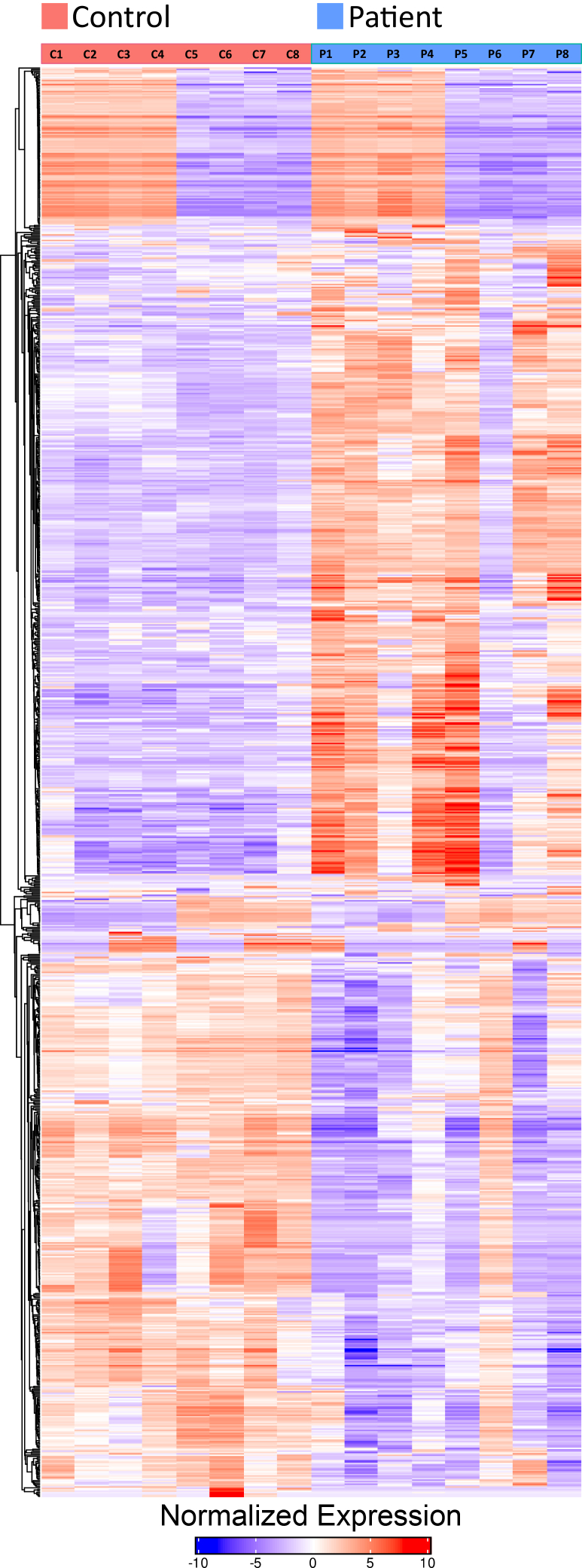
**Unsupervised clustering analysis revealed a clear separation between PG and control samples in human.** n = 8 for each group. PG, pyoderma gangrenosum.

**Figure 3:**
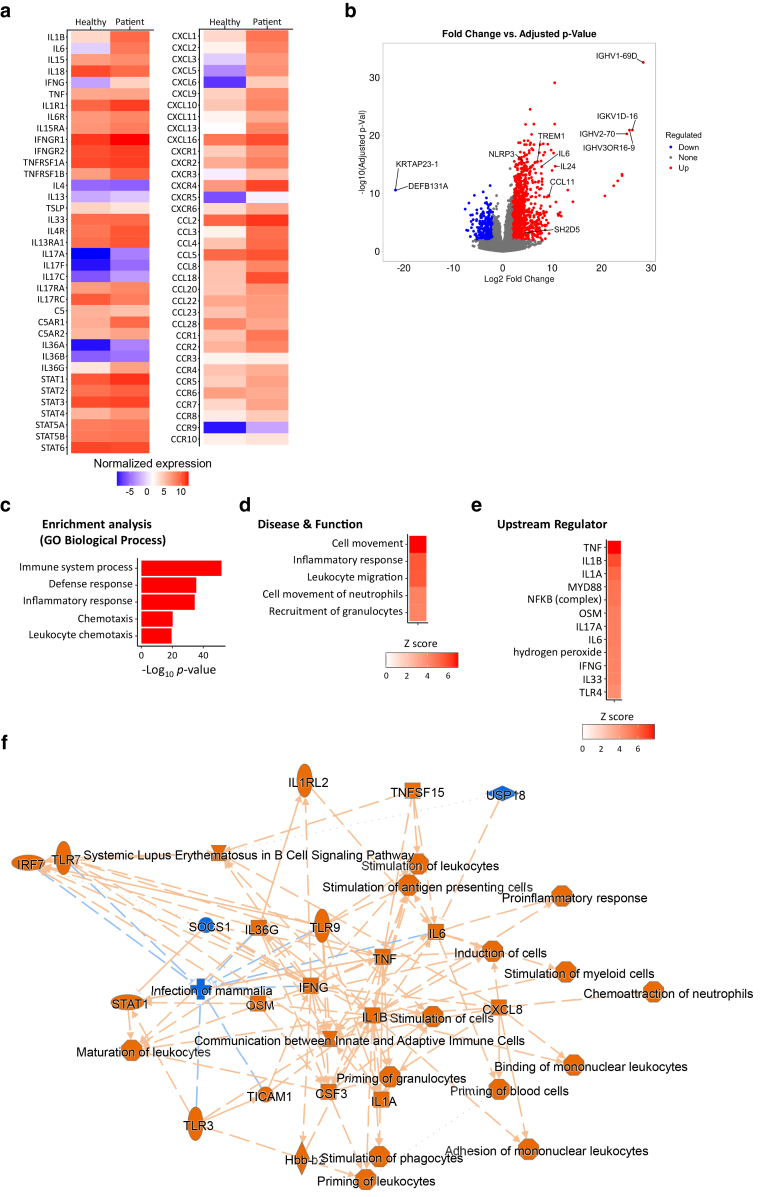
**Transcriptomic profiling of lesional skin from patients with PG.** (**a**) Relative mRNA expression levels of inflammation-associated genes in lesional skin from patients with PG compared with that in healthy control skin. Expression values are shown as log_2_-transformed count values. n = 8 per group. (**b**) Volcano plot showing differentially expressed genes between PG lesional skin and healthy control skin. n = 8 per group. (**c**) GO biological process terms enriched among genes upregulated in PG lesional skin compared with those in healthy control skin. (**d, e**) IPA of RNA-sequencing data comparing PG lesional skin and healthy control skin: (**d**) diseases and functions terms related to cell movement, inflammatory response, and leukocyte migration identified among differentially expressed genes, and (**e**) upstream regulator analysis predicting activation of *TNFα*, *IL1β*, *IL1α*, *OSM*, *IL17*, and *IL6*. (**f**) Network analysis revealed close interactions among proinflammatory cytokines and chemokines, which is a key mediator of neutrophil recruitment and activation. Orange lines (or arrows) indicate activation, and blue lines (or arrows) indicate inhibition of biological pathways, molecules, or functions. GO, Gene Ontology; IPA, ingenuity pathway analysis; PG, pyoderma gangrenosum.

To investigate the molecular inflammatory signature of human PG, we performed transcriptomic analysis using a published RNA-sequencing dataset comparing lesional skin from patients with PG with healthy control skin. Unsupervised clustering analysis revealed a clear separation between PG and control samples, indicating substantial differences in global gene expression profiles (Figure 2). Analysis of cytokine- and chemokine-related gene expression demonstrated marked upregulation of inflammatory mediators in PG lesions, with a predominant enrichment of type 1–associated cytokines and chemokines, such as *IFNγ*, *IL1β*, *CXCL9*, *CXCL10*, and *CXCL11* (Figure 3a). Volcano plot analysis identified the upregulation of various proinflammatory cytokines and immune-related genes in PG. Among the upregulated genes, Ig-related families exhibited the largest fold changes, whereas *KRTAP23-1* (a hair keratin-associated protein) and *DEFB131A* (β-defensin) showed the greatest fold changes among the downregulated genes (Figure 3b). Gene Ontology enrichment analysis of the genes upregulated in PG (patient/control: fold change > 4, *P* < .01) revealed strong enrichment of biological processes related to immune system processes, defense response, inflammatory response, chemotaxis, and leukocyte chemotaxis (Figure 3c). Consistent with these findings, Ingenuity Pathway Analysis demonstrated activation of functional networks associated with cell movement, inflammatory response, leukocyte migration, and recruitment of granulocytes **(**Figure 3d). Furthermore, upstream regulator analysis identified increased activation scores for proinflammatory cytokines, including *TNFα*, *IL1β*, *IL1α*, *OSM*, *IL17A*, *IL-6*, and *IFNγ* (Figure 3e). Network analysis revealed close interactions among these cytokines and chemokines, which are key mediators of neutrophil recruitment and activation (Figure 3f). Taken together, these results indicate that lesional skin in patients with PG is characterized by enhanced type 1 inflammatory responses and activation of neutrophil-associated inflammatory pathways.

### Development of a modified brequinar-induced murine model of PG

**Figure 4:**
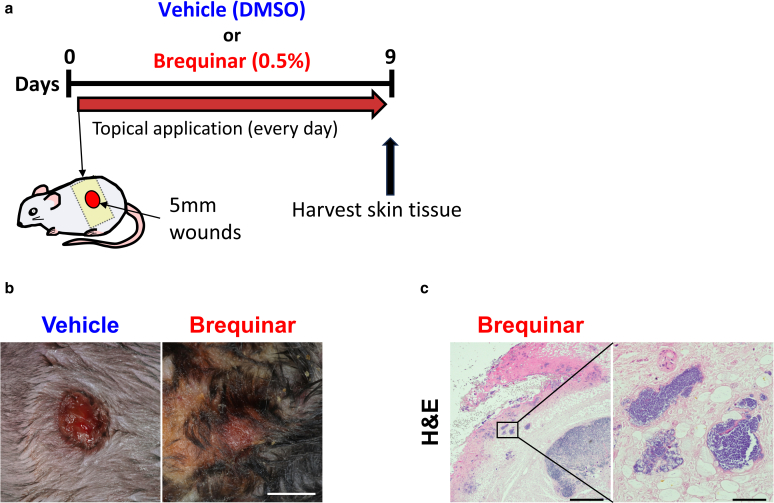
**Previously reported topical brequinar-induced PG model.(a)** Schematic representation of the previously reported murine PG-like model generated by topical application of brequinar (0.5%) or DMSO as a vehicle control to full-thickness excisional wounds for 9 consecutive days. Skin tissues were harvested on day 9 after wounding. (**b**) Representative photographs to show erosion and erythema around the wound of mice treated with 0.5% brequinar compared with that of vehicle-treated controls. (**c**) Representative H&E staining showing extensive inflammation and necrosis within the wound bed accompanied by massive aggregation of cocci in PG compared with that in healthy control skin. PG, pyoderma gangrenosum. Scale bar (**b**) = 3 mm. Scale bar panel **c** left panel = 500 μm; right panel = 50 μm.

Next, we examined the effect of topical application of brequinar on wound healing in mice. On the basis of a previously reported protocol (Jatana et al, 2023), 0.5% brequinar or DMSO as a vehicle control was formulated with petroleum jelly (Figure 4a). They were applied topically to full-thickness excisional wounds once daily for 9 consecutive days. Mice treated with 0.5% brequinar exhibited erosion and erythema around the wound area compared with vehicle-treated controls (Figure 4b). However, histopathological analysis revealed extensive inflammation and necrosis within the wound bed, accompanied by massive aggregation of cocci, indicating secondary bacterial infection (Figure 4c). Such bacterial colonization was not observed in vehicle-treated mice and was not improved by wound covering during the observation period. To minimize the contribution of secondary bacterial infection to local inflammatory responses, mice were treated with enrofloxacin, a broad-spectrum antibiotic, administered in drinking water throughout the experimental period. Under conditions of effective bacterial infection control achieved by antibiotic treatment, topical application of 0.5% brequinar modestly impaired wound healing and induced type 1 inflammatory cytokine expression compared with observation in vehicle-treated wounds (data not shown).

**Figure 5:**
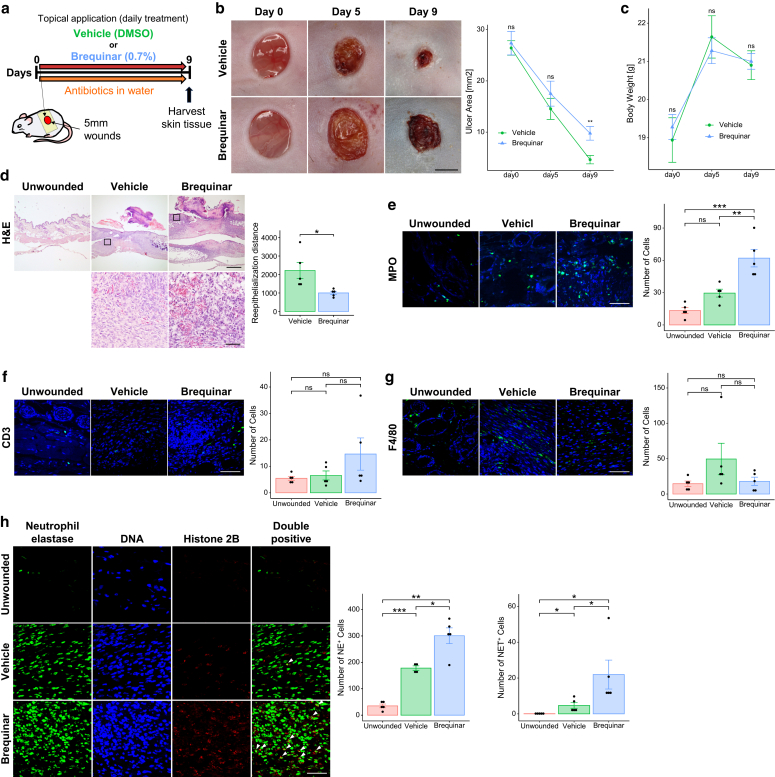
**Development of a modified brequinar-induced murine model of PG.** (**a**) Schematic representation of the murine PG-like model generated by topical application of brequinar (0.7%) or DMSO as a vehicle control to full-thickness excisional wounds for 9 consecutive days. Antibiotics were administered in drinking water throughout the experimental period. Skin tissues were harvested on day 9 after wounding. (**b**) Representative photographs of wound areas in vehicle- and brequinar-treated mice at days 0, 5, and 9 after wounding. Quantification of wound area is shown in the right panel and was performed using ImageJ software. n = 8 wounds per group. Bar = 3 mm. (**c**) Body weight changes in vehicle- and brequinar-treated mice measured at days 0, 5, and 9 after wounding. (**d**) Representative H&E-stained images showing increased inflammatory cell infiltration and expanded necrotic areas in wounds from brequinar-treated mice compared with those in unwounded skin and vehicle-treated wounds. Quantification of re-epithelialization distance is shown in the right panel and was performed using ImageJ software. n = 5 per group. Bar = 50 μm. (**e–g**) Representative immunofluorescence images showing (**e**) MPO^+^ neutrophils, (**f**) CD3^+^ T cells, and (**g**) CD68^+^ macrophages in unwounded skin and wound areas from vehicle- or brequinar-treated mice. Quantification of positive cells is shown in the right panel and was performed by counting cells in 4 randomly selected fields per section. n = 5 per group. Bar = 50 μm. (**h**) Representative immunofluorescence images showing NET formation in unwounded skin and wound areas from vehicle- or brequinar-treated mice. Neutrophil elastase (green), DNA (blue), and histone H2B (red) are shown. Areas positive for both neutrophil elastase and histone H2B indicate NET formation. Quantification is shown in the right panel and was performed by counting positive cells in 4 randomly selected fields per section. n = 5 per group. Bar = 50 μm. MPO, myeloperoxidase; NET, neutrophil extracellular trap; n.s., not significant; PG, pyoderma gangrenosum.

To induce a more robust and reproducible PG-like inflammatory phenotype, we increased the concentration of brequinar to 0.7%, dissolved it in warmed DMSO, and formulated it with petroleum jelly (Figure 5a). Treatment with 0.7% brequinar resulted in significantly delayed wound closure, with wound areas remaining significantly larger than those of vehicle-treated mice on days 5 and 9 after wounding (Figure 5b). There was no difference in body weight between the 2 groups, indicating the absence of overt systemic toxicity (Figure 5c). Histopathological study using H&E staining revealed an increased area of necrosis in the central region of the wound bed in brequinar-treated mice, without evidence of bacterial aggregation; quantitative analysis revealed a longer re-epithelialization distance in brequinar-treated mice (Figure 5d). Immunofluorescence staining revealed that the number of infiltrating MPO^+^ neutrophils was increased in the wounded area of brequinar-treated mice compared with that in vehicle-treated wounds and unwounded normal skin (Figure 5e). In contrast, the numbers of infiltrating CD3^+^ T cells and CD68^+^ macrophages did not differ among the groups (Figure 5f and g). Consistent with findings in lesional skin from patients with PG, NE^+^ neutrophils and NET formation were significantly increased in brequinar-treated wounds (Figure 5h). Taken together, these results indicate that optimized topical application of brequinar induces neutrophil activation and prominent NET formation in wounded skin, closely recapitulating key pathological features of human PG.

### Brequinar-induced PG-like lesions exhibit enhanced proinflammatory and chemokine expressions

**Figure 6:**
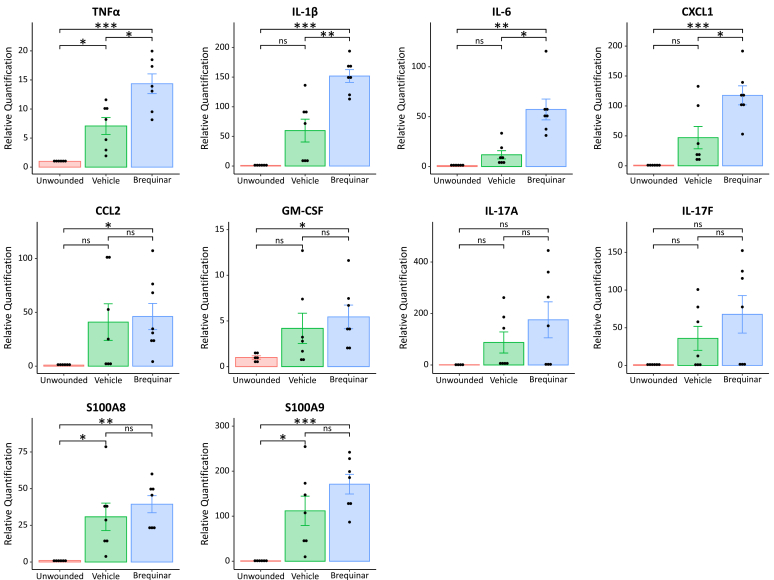
**Enhanced proinflammatory cytokines and chemokine expressions in the brequinar-induced murine model of PG.** Relative mRNA expression levels of proinflammatory cytokines, chemokines, and S100 family proteins in unwounded skin and wound tissue from vehicle- or brequinar-treated mice at day 9 after wounding are shown. n = 6 for unwounded skin and n = 7 for vehicle- and brequinar-treated groups. Data are expressed as mean ± SEM. ∗∗∗*P* < .001, ∗∗*P* < .01, and ∗*P* < .05. PG, pyoderma gangrenosum.

To clarify the inflammatory profile induced by brequinar, we examined the expression of proinflammatory cytokines and chemokines in wounded skin at day 9 after wounding. qPCR analysis demonstrated that topical brequinar application enhanced the mRNA expression of type 1–associated proinflammatory cytokines, including *TNFα*, *IL1β*, and *IL6* compared with vehicle treatment. Moreover, chemokines involved in leukocyte recruitment, such as *CXCL1*, *CCL2*, and *GM-CSF*, were significantly upregulated in brequinar-treated wounds compared with those in unwounded skin (Figure 6). In addition, the expression of neutrophil-associated inflammatory markers *S100A8* and *S100A9* was increased in brequinar-treated wounds. In contrast, *IL17A* and *IL17F* expressions did not show statistically significant differences among the groups. These results indicate that topical brequinar application enhances type 1 inflammatory responses in the wound area. This excessive inflammation might induce necrosis and an intractable wound-healing process, mimicking human PG.

### Enhanced oxidative stress and activation of Nrf2 signaling in human PG and brequinar-induced murine lesions

**Figure 7:**
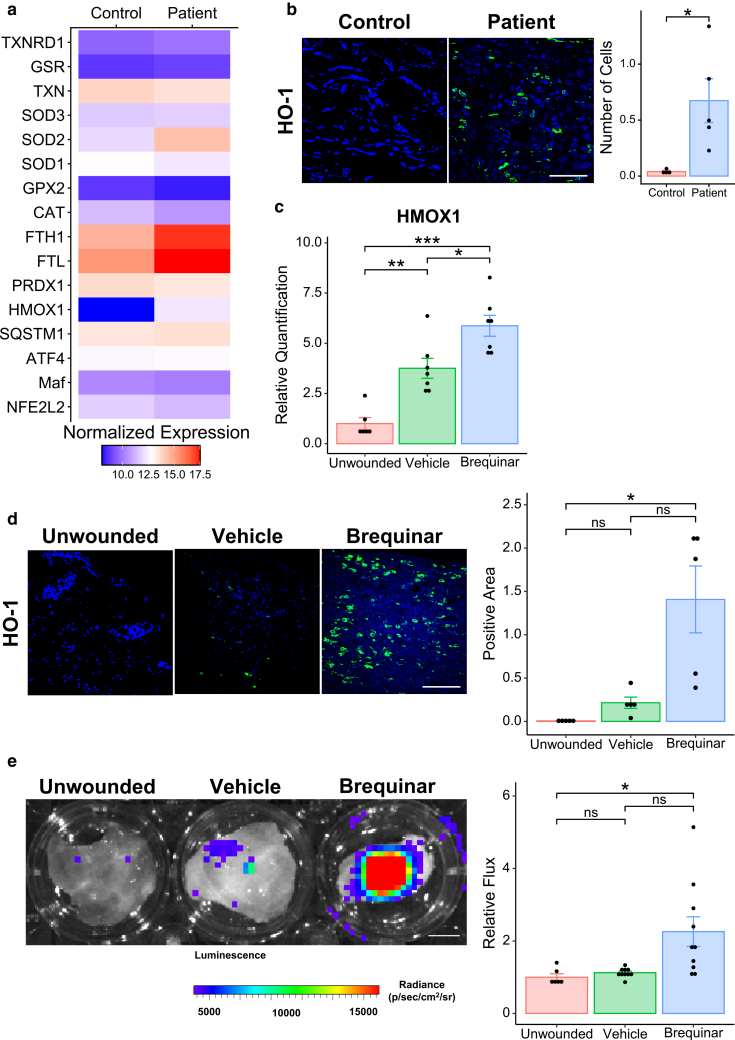
**Enhanced oxidative stress and activation of Nrf2 signaling in human PG and brequinar-induced murine lesions.** (**a**) Relative mRNA expression levels of Nrf2 downstream target genes identified by IPA in lesional skin from patients with PG compared with those in healthy control skin. Expression values are shown as log_2_-transformed count values. n = 8 per group. (**b**) Representative immunofluorescence images showing HO-1^+^ areas in lesional skin from patients with PG compared with those in healthy control skin. Quantification of HO-1^+^ areas is shown in the right panel and was performed in 6 randomly selected fields per section. n = 5 per group. Bar = 50 μm. (**c**) Relative mRNA expression levels of *HMOX1* in unwounded skin and wound tissue from vehicle- or brequinar-treated mice at day 9 after wounding. n = 6 for unwounded skin and n = 7 for vehicle- and brequinar-treated groups. (**d**) Representative immunofluorescence staining images for HO-1^+^ area in unwounded and wound area in vehicle- or brequinar-treated mice. Right graph shows the quantification of positive area counted in 4 randomized area in each slide. n = 5 for each group. Bar = 50 μm. (**e**) Representative images of OKD-Luc signal in unwounded skin and wound areas from vehicle- or brequinar-treated mice. Quantification of OKD-Luc signal intensity is shown in the right panel, with values normalized to those of unwounded skin (set to 1). n = 6 for unwounded skin and n = 10 for vehicle- and brequinar-treated groups. Bar = 5 mm. Data are expressed as mean ± SEM. ∗∗∗*P* < .001, ∗∗*P* < .01, and ∗*P* < .05. HO-1, heme oxygenase-1; IPA, ingenuity pathway analysis; PG, pyoderma gangrenosum.

NET formation is tightly linked to oxidative stress, and disrupted reduction–oxidation regulation has been reported in patients with PG (Georgescu et al, 2025; Kirchner et al, 2012). On this basis, we investigated the potential involvement of oxidative stress in PG pathogenesis. Transcriptomic analysis of RNA-sequencing datasets from human PG lesions and healthy control skin revealed significant upregulation of genes downstream of the Nrf2 pathway, including *HMOX1*, *SOD2*, and *FTL*, in PG lesions (Figure 7a). Immunostaining demonstrated increased expression of heme oxygenase-1 in lesional skin from patients with PG compared with that in normal control skin (Figure 7b). In the brequinar-induced murine model, both heme oxygenase-1 mRNA and protein levels were significantly increased in brequinar-treated wounds compared with those in unwounded skin and vehicle-treated wounds, as assessed by qPCR and immunostaining, respectively (Figure 7c and d). To assess activation of Nrf2 signaling in vivo, we utilized OKD-Luc (B6-ARE-NRF2/LUC) reporter mice, in which luciferase expression reflects Nrf2-dependent transcriptional activity. Results revealed that Nrf2 reporter activity was significantly elevated at wound sites in brequinar-treated mice compared with that in vehicle-treated mice and unwounded skin (Figure 7e). These results demonstrate that enhanced oxidative stress responses accompanied by activation of Nrf2 signaling are observed in both human and mouse PG models, supporting a possible contribution of oxidative stress–associated pathways in PG pathogenesis.

### Transcriptome analysis revealed similar enhancement of inflammatory genes expression in brequinar-induced PG mouse model

**Figure 8:**
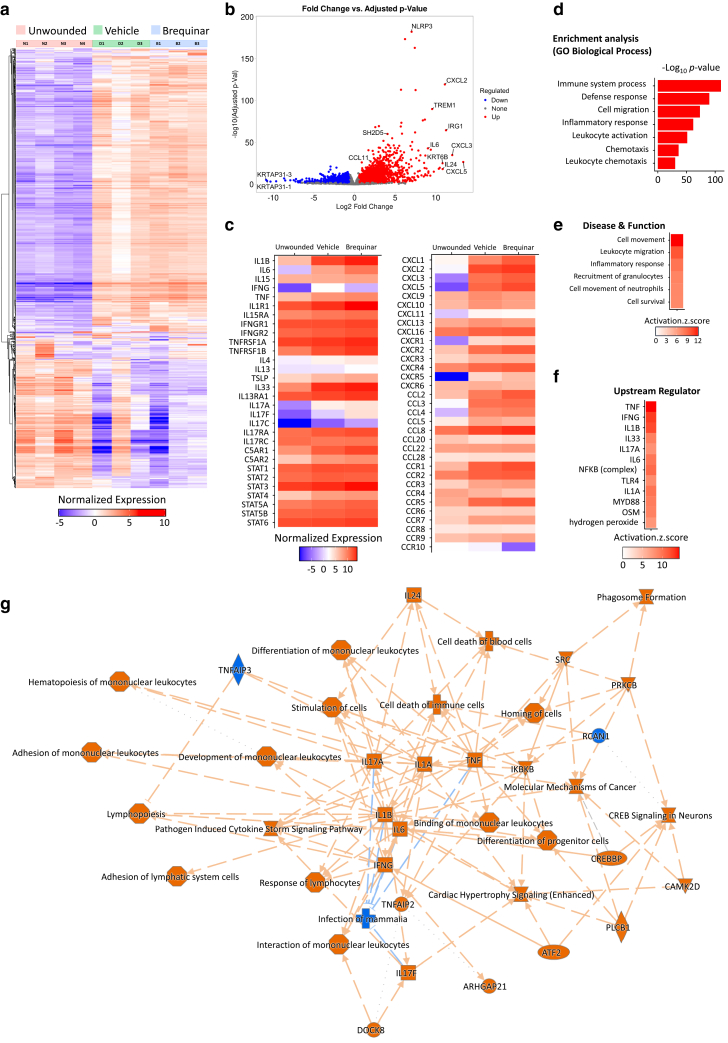
**Transcriptome analysis revealed similar enhancement of inflammatory genes expression in brequinar-induced PG mouse model.** (**a**) Unsupervised clustering analysis of RNA-sequencing gene expression profiles from unwounded skin and wounded skin at day 9 after wounding in vehicle-treated and brequinar-treated mice. (**b**) Volcano plot showing differentially expressed genes between brequinar-treated wounds and unwounded skin. **(c)** Relative mRNA expression levels of inflammation-associated genes in unwounded skin and wounded skin in vehicle-treated and brequinar-treated mice at day 9. Expression values are shown as log_2_-transformed count values. n = 4 for unwounded skin and n = 3 for vehicle- and brequinar-treated groups. (**d**) GO biological process terms enriched among genes upregulated in brequinar-treated mice compared with those in unwounded skin. (**e, f**) IPA analysis of RNA-sequencing data in the brequinar-induced murine PG model and unwounded skin: (**e**) diseases and functions terms related to immune system processes, defense responses, cell migration, and inflammatory responses and (**f**) upstream regulator analysis predicting activation of *TNFα*, *IFNγ*, *IL1β*, *IL1α*, *IL6*, *OSM*, and *IL17A*. (**g**) Network pathway analysis demonstrates the interactions among proinflammatory cytokines and leukocytes activation in PG skin lesions in mouse. Orange lines (or arrows) indicate activation, and blue lines (or arrows) indicate inhibition of biological pathways, molecules, or functions. GO, Gene Ontology; IPA, ingenuity pathway analysis; PG, pyoderma gangrenosum.

**Figure 9:**
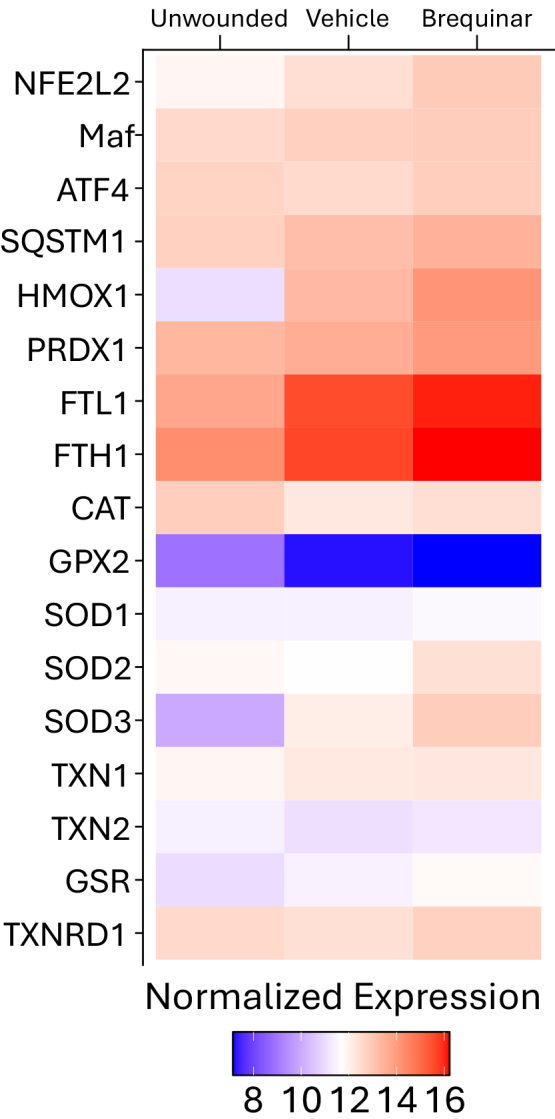
**Unsupervised clustering analysis revealed a clear separation between PG and control samples in human.** n = 8 for each group. PG, pyoderma gangrenosum.

To characterize global gene expression patterns in the brequinar-induced PG mouse model, RNA-sequencing analysis was performed using skin samples obtained from unwounded skin, vehicle-treated wounds, and brequinar-treated wounds. Unsupervised clustering analysis revealed that wounding itself induced substantial alterations in gene expression compared with unwounded skin (Figure 8a). Volcano plot analysis comparing brequinar-treated wounds with unwounded skin identified upregulation of inflammatory and immune-related genes (fold change > 1.5, *P* < .01). Among these, inflammatory chemokines such as *CXCL2*, *CXCL3*, and *CXCL5* were among the most prominently upregulated, whereas keratin-associated protein genes, including *KRTAP31-3* and *KRTAP31-1*, were significantly downregulated (Figure 8b). We next examined curated gene sets related to T helper (Th)1, Th2, and Th17 cytokines; signal transducer and activator of transcription family signaling; and chemokines. This analysis demonstrated broad induction of proinflammatory cytokines and chemokines in brequinar-treated wounds, with a marked predominance of Th1-associated signatures (Figure 8c). Moreover, genes downstream of the Nrf2 pathway, including *HMOX1*, *SOD2*, *SOD3*, and *FTL*, were upregulated in PG mouse skin lesions (Figure 9).

Gene Ontology enrichment analysis of the genes upregulated in brequinar-treated wounds demonstrated significant enrichment of biological processes related to immune system process, defense response, cell migration, inflammatory response, leukocyte activation, and chemotaxis compared with those in unwounded skin (Figure 8d). Ingenuity Pathway Analysis revealed activation of functional networks associated with cell movement, leukocyte migration, and inflammatory responses and recruitment of granulocytes (Figure 8e). Moreover, upstream regulator analysis further identified increased activation of key proinflammatory cytokines, including *TNFα*, *IFNγ*, *IL1β*, *IL1α*, *IL6*, and *OSM* (Figure 8f). Network pathway analysis also showed the relationships among these cytokines (Figure 8g). Taken together, these transcriptomic analyses demonstrate that enhanced Th1-dominant inflammation and neutrophil-associated inflammatory signaling are prominent features of lesional skin in the brequinar-induced PG mouse model, closely recapitulating the inflammatory landscape observed in human PG.

### Activation of the NLRP3 inflammasome and C5a signaling in human PG and brequinar-induced murine PG model

**Figure 10:**
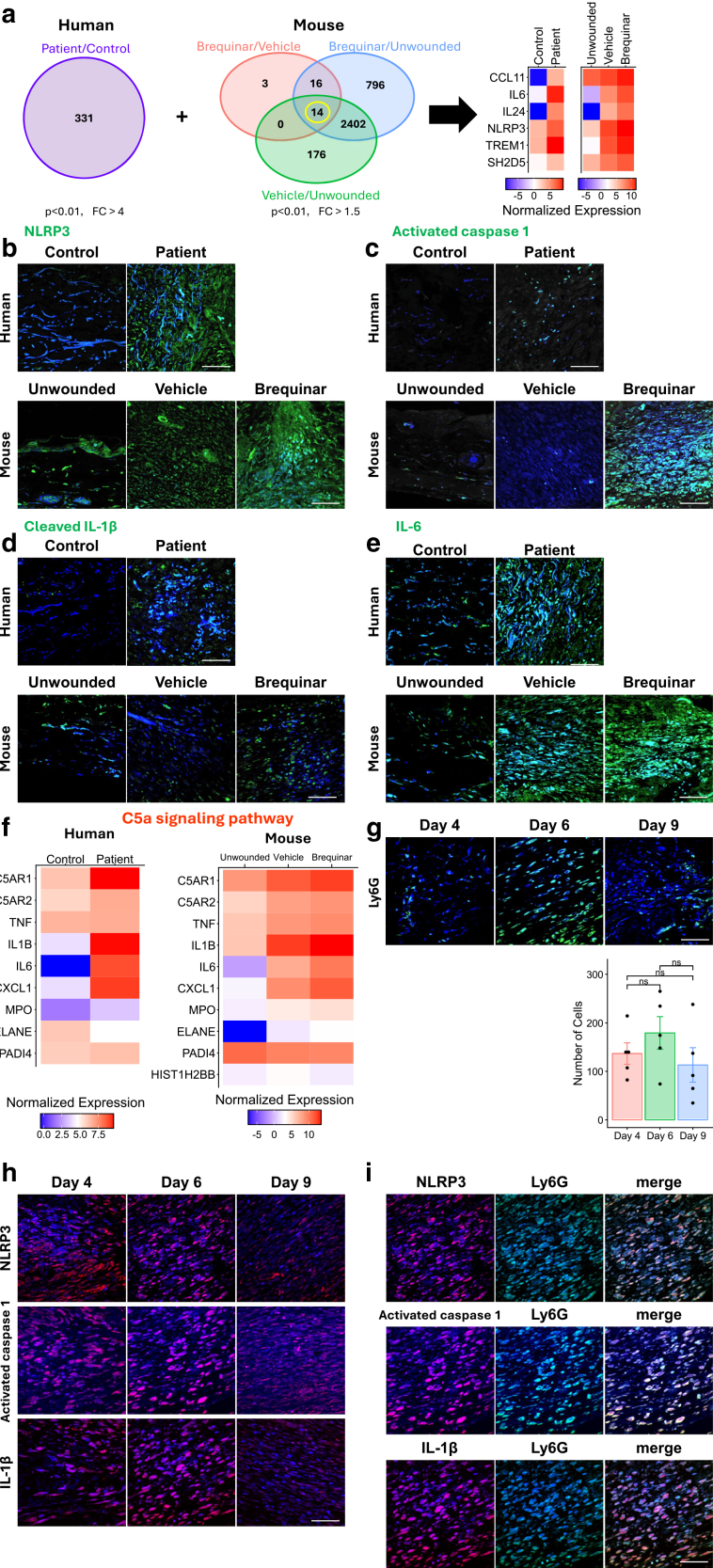
**Activation of the NLRP3 inflammasome and C5a signaling in human and brequinar-induced murine PG model.** (**a**) Venn diagram summarizing the comparison of upregulated genes identified by transcriptomic analyses. The left panel shows genes upregulated in PG lesional skin compared with those in normal skin in human samples (purple; 331 genes). The center panel shows genes upregulated in murine samples under the following comparisons: brequinar-treated wounds versus vehicle-treated wounds (red), brequinar-treated wounds versus unwounded skin (blue), and vehicle-treated wounds versus unwounded skin (green). Thirty-one genes were commonly upregulated across these murine comparisons. The right panel shows relative mRNA expression levels of 6 genes identified as commonly upregulated in both human PG (331 genes) and the murine PG model (14 genes). (**b–e**) Representative immunofluorescence images showing (**b**) NLRP3, (**c**) activated caspase-1, (**d**) cleaved IL-1β, and (**e**) IL-6 expression in healthy control skin and PG lesional skin from human samples and in unwounded skin as well as vehicle-treated and brequinar-treated wounds from mouse samples. n = 4 per group. Bar = 50 μm. **(f)** Relative mRNA expression levels of C5a signaling pathway–related genes in lesional skin from patients with PG and healthy control skin and in unwounded skin and day 9 wounds from vehicle-treated and brequinar-treated mice. Expression levels are presented as log_2_-transformed normalized count values. n = 8 per group for human samples, n = 4 for unwounded skin, and n = 3 each for the vehicle-treated and brequinar-treated groups. **(g)** Representative immunofluorescence images showing Ly6G^+^ cells in brequinar-treated wounds on day 4, 6, and 9 after injury, along with quantification of the number of positive cells. n = 5 for each group. Bar = 50 μm. **(h)** Representative immunofluorescence images showing NLRP3, activated caspase-1, and cleaved IL-1β expression in brequinar-treated wounds from mouse samples. Bar = 50 μm. **(i)** Representative immunofluorescence images showing coimmunostaining of Ly6G with NLRP3, activated caspase-1, and cleaved IL-1β in brequinar-treated wounds from mouse samples. Bar = 50 μm. PG, pyoderma gangrenosum.

To identify the molecular and immunological mechanisms shared between human PG and the brequinar-induced murine model, we performed an integrative cross-species transcriptomic analysis (Figure 10a). Comparison of human PG lesional skin with normal skin identified 331 genes that were significantly upregulated in PG lesions (purple). In the murine dataset, differential expression analyses focusing on upregulated genes identified 33 genes that were upregulated in brequinar-treated wounds compared with those in vehicle-treated wounds (red), 3228 genes that were upregulated in brequinar-treated wounds compared with those in unwounded skin (blue), and 2592 genes that were upregulated in vehicle-treated wounds compared with those in unwounded skin (green). Intersection of these gene sets identified 14 genes whose expression was induced by wounding and subsequently further increased in brequinar-treated wounds compared with those in vehicle-treated wounds. Cross-referencing the human and murine datasets further identified 6 genes that were commonly upregulated in both human PG lesions and the brequinar-induced mouse model (Figure 10a). Among these genes, NLRP3 forms an inflammasome complex that activates caspase-1, leading to IL-1β maturation and subsequent amplification of type 1 inflammatory responses, including increased IL-6 expression (Cambon et al, 2024; Martinon et al, 2002). Immunofluorescence analysis showed increased expression of NLRP3 and activated caspase-1 in lesional skin from patients with PG compared with that in normal human skin as well as in brequinar-treated murine wounds compared with that in unwounded skin and vehicle-treated wounds (Figure 10b and c). In parallel, increased expression of cleaved IL-1β and IL-6 was observed in both human PG and murine lesions (Figure 10d and e).

We further explored potential upstream inflammatory signals associated with NLRP3 activation. Transcriptomic analyses revealed the upregulation of multiple genes related to the C5a signaling pathway in both human PG lesions and brequinar-induced murine wounds (Figure 10f), suggesting a possible contribution of C5a-associated inflammatory signaling to inflammasome activation and neutrophil responses.

We further examined the temporal sequence of inflammatory events by immunofluorescence staining of lesional skin harvested on days 4, 6, and 9 after brequinar administration. Ly6G^+^ cells showed a mild tendency to increase on day 6 compared with that on day 4 and reached a peak on day 9 (Figure 10g). Immunohistochemical analysis demonstrated that NLRP3, cleaved caspase-1, and IL-1β were strongly expressed on days 4 and 6, whereas their expression was relatively reduced on day 9 (Figure 10h). Notably, Ly6G^+^ neutrophils exhibited enhanced expression of all 3 inflammasome-related markers (Figure 10i), suggesting that infiltrating neutrophils might be a major cellular source of NLRP3 inflammasome activation in this model.

Taken together, these findings suggest that NLRP3 inflammasome activation is associated with inflammatory responses in both human PG and the brequinar-induced PG-like mouse model.

### Therapeutic effects of prednisolone and C5a pathway inhibition in the brequinar-induced PG mouse model

**Figure 11:**
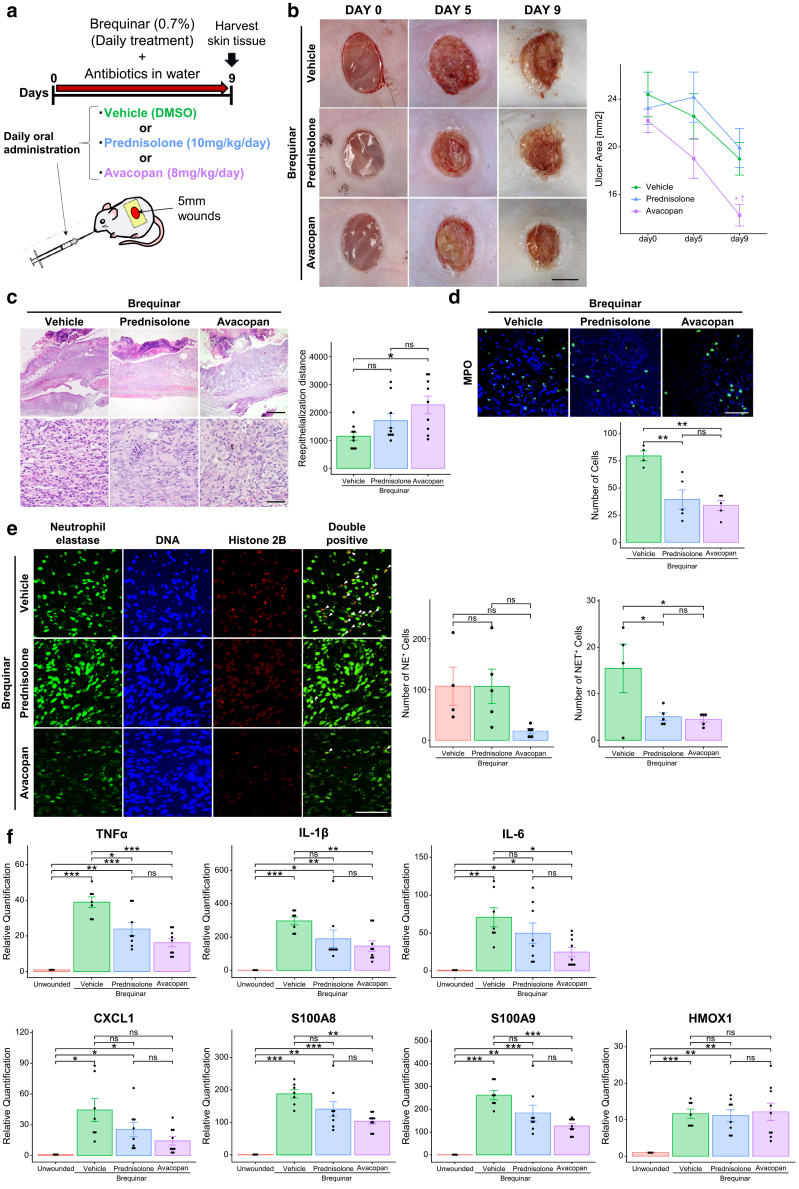
**Therapeutic effects of prednisolone and C5a pathway inhibition in the brequinar-induced PG mouse model.** (**a**) Schematic representation of the treatment protocol. Prednisolone, avacopan, or methyl cellulose as a vehicle control was administered orally once daily for 9 consecutive days in the brequinar-induced PG mouse model under antibiotics treatment. Skin tissues were harvested on day 9 after wounding. (**b**) Representative photographs of wound areas in vehicle-, prednisolone-, and avacopan-treated mice at days 0, 5, and 9 after wounding. Bar = 3 mm. Quantification of wound area is shown in the right panel. n = 8 wounds per group. ∗*P* < .05 (vehicle vs avacopan) and **†***P* < .05 (prednisolone vs avacopan). (**c**) Representative H&E-stained image showing reduced inflammatory cell infiltration and necrotic areas in wounds from prednisolone- and avacopan-treated mice compared with those in vehicle-treated controls in the brequinar-induced PG model. Quantification of re-epithelialization distance is shown in the right panel and was performed using ImageJ software. n = 4 for vehicle and n = 5 for prednisolone and avacopan groups. Bar = 500 μm (upper panels) and 50 μm (lower panels). (**d**) Representative immunofluorescence images showing MPO^+^ neutrophils in wound tissue from vehicle-, prednisolone-, and avacopan-treated mice. Quantification of positive cells is shown in the right panel and was performed by counting cells in 4 randomly selected fields per section. n = 4 for vehicle and n = 5 for prednisolone and avacopan groups. Bar = 50 μm. (**e**) Representative immunofluorescence images showing NET formation in wound tissue from vehicle-, prednisolone-, and avacopan-treated mice. Neutrophil elastase (green), DNA (blue), and histone H2B (red) are shown. Areas positive for both neutrophil elastase and histone H2B indicate NET formation. Quantification is shown in the right panel and was performed by counting positive areas in 4 randomly selected fields per section. n = 4 for vehicle and n = 5 for prednisolone and avacopan groups. Bar = 50 μm. (**f**) Relative mRNA expression levels of proinflammatory cytokines, chemokines, and S100 family genes in unwounded skin and wound tissue from each treatment group at day 9 after wounding. n = 3 for unwounded skin, n = 7 for vehicle, n = 9 for avacopan, and n = 8 for prednisolone groups. Data are expressed as mean ± SEM. ∗∗∗*P* < .001, ∗∗*P* < .01, and ∗*P* < .05. MPO, myeloperoxidase; NET, neutrophil extracellular trap; PG, pyoderma gangrenosum.

On the basis of our preceding findings demonstrating that the brequinar-induced PG mouse model shares key inflammatory signaling pathways with human PG, we next evaluated whether this model also responds to therapeutic interventions commonly used or proposed for PG. Systemic corticosteroids are widely used as a first-line therapy for PG in clinical practice. Recently, increasing attention has been directed toward therapeutic inhibition of the complement C5a pathway for various neutrophil-associated diseases, including thrombosis, vasculitis, and PG (Chen et al, 2022; Shiratori-Aso and Nakazawa, 2023; Wang et al, 2024). We therefore examined the therapeutic potential of prednisolone and avacopan, a selective C5a receptor antagonist, in the brequinar-induced PG mouse model (Figure 11a).

Avacopan treatment resulted in a significant improvement in wound healing in brequinar-induced PG model mice, as evidenced by a reduced wound area compared with that in both vehicle- and prednisolone-treated mice on day 9 after wounding. In contrast, prednisolone treatment did not lead to a significant improvement in wound closure compared with vehicle treatment (Figure 11b). Histopathological examination revealed reduced inflammatory cell infiltration within the wound bed in both prednisolone- and avacopan-treated mice compared with that in vehicle-treated controls. However, no significant difference was observed between the vehicle and prednisolone groups. In contrast, the avacopan group exhibited a significantly greater re-epithelialization length than the vehicle group (*P* = .01) (Figure 11c). Immunostaining analysis showed that the numbers of infiltrating MPO^+^ or NE^+^ neutrophils and the extent of NET formation were significantly decreased in the avacopan-treated group relative to those in vehicle-treated wounds. In the prednisolone-treated group, a similar trend was observed; however, no significant difference in NE^+^ cells was detected compared with controls (Figure 11d and e). qPCR analysis demonstrated that brequinar-induced upregulation of type 1 inflammation–associated genes, including *TNFα*, *IL1β*, and *IL-6*, was significantly suppressed by both prednisolone and avacopan treatments (Figure 11f). Notably, avacopan tended to exert stronger suppressive effects on inflammatory gene expression than prednisolone, although these differences did not reach statistical significance. In contrast, expression levels of *HMOX1* were not significantly altered by either treatment. Taken together, these results demonstrate that the brequinar-induced PG mouse model responds to therapeutic interventions relevant to human PG and that type 1 inflammation and neutrophil activation are key contributors to disease pathology in this model.

## Discussion

In this study, we conducted a comprehensive comparative analysis of human PG and a brequinar-induced murine PG model by integrating histopathological, immunological, transcriptomic, and therapeutic response analyses. Brequinar is a potent inhibitor of dihydroorotate dehydrogenase, a key enzyme in the de novo biosynthesis of pyrimidines that catalyzes the conversion of dihydroorotate to orotate, thereby potentially depleting cellular pyrimidine nucleotides required for RNA and DNA synthesis (Chen et al, 1986; Peters et al, 1987; Schwartsmann et al, 1988; Sykes et al, 2016). During wound healing, it may exacerbate inflammatory responses and delay tissue repair, including decreased wound closure and delayed re-epithelialization, thereby creating a permissive environment for PG-like pathology. Previous studies investigating N-phosphonacetyl-L-aspartate– and brequinar-induced PG-like mouse models have reported systemic effects, including body weight loss and intestinal inflammation, and have discussed potential links between these models and immune dysregulation associated with inflammatory bowel disease (Jatana et al, 2023). In contrast, in our modified brequinar-induced PG mouse model, no significant body weight loss was observed in either the control or brequinar-treated groups throughout the observation period. These differences from previous reports may be attributable to variations in experimental conditions, such as drug dosage or infection control. Prophylactic antibiotics were administered to prevent secondary bacterial infection related to topical brequinar application. Consistent with this approach, clinical evidence suggests that antibiotic monotherapy has limited efficacy in PG. In a Korean single-center retrospective study, only 2 of 12 patients who received antibiotics as initial treatment achieved complete or partial remission (Kim et al, 2023). On the basis of these considerations, we adopted an experimental approach in which the brequinar-induced PG model was evaluated under conditions that controlled for the potential confounding effects of secondary infection. However, we cannot exclude the possibility that continuous enrofloxacin treatment influenced skin inflammation, microbiota composition, neutrophil activation, or wound-healing responses, and these potential effects should be considered when interpreting the findings of this study.

The mechanisms underlying NLRP3 activation in PG are likely multifactorial. One plausible upstream trigger is oxidative stress, which is generated during tissue injury and excessive inflammation in various types of intractable wounds (Wang et al, 2025; Wlaschek and Scharffetter-Kochanek, 2005). In support of this notion, we detected activation of the Nrf2 pathway in the skin site in both human PG and mouse PG model, indicating increased oxidative stress at lesional skin sites. ROS are well-established activators of the NLRP3 inflammasome, and the ROS–NLRP3 axis can form a self-sustaining loop that drives persistent oxidative stress and inflammation in diseased skin (Dominic et al, 2022; Harijith et al, 2014). Upstream regulator analysis supported the activation of NF-κB–associated inflammatory signaling, with the predicted involvement of TNF-α and IL-1 family cytokines, which are well-established drivers of neutrophil recruitment, NET formation, and amplification of inflammatory responses (Barnabei et al, 2021; Cavalli et al, 2021; Cowburn et al, 2004; Keshari et al, 2012). Together, these pathways may converge to promote sustained inflammasome activation, resulting in excessive cytokine production and tissue damage characteristic of PG.

Another important aspect of PG pathogenesis highlighted by our study is the dominant role of neutrophils and NET formation. Histological and immunofluorescence analyses demonstrated a marked increase in MPO^+^ neutrophils and NET in PG lesions, whereas CD3^+^ T-cell infiltration was not significantly elevated in either human samples or the mouse model. These findings support the concept that PG is primarily driven by dysregulated innate immune responses rather than adaptive immunity. Although T cells are known to contribute to PG pathogenesis, as supported by the clinical efficacy of cyclosporine, our data suggest that neutrophil-mediated inflammation may represent the predominant effector mechanism, particularly in the active ulcerative phase. However, the lack of temporal analysis of T-cell dynamics represents a limitation of this study and warrants further investigation. Macrophages play multiple roles at sites of cutaneous injury, including the production of proinflammatory cytokines and tissue repair (Krzyszczyk et al, 2018). It has been reported that there are increased CD163^+^ macrophages in the peripheral area of lesional skin in patients with PG and a possible association with the local immune environment (Alavi et al, 2017; Marzano et al, 2010). In our analysis, CD68^+^ macrophages were increased in human PG lesions compared with that in normal skin; however, F4/80^+^ macrophages did not increase in the mouse PG model. This discrepancy may reflect species-specific differences, differences in disease stage, or functional heterogeneity of macrophage subsets. Moreover, given the dual roles of macrophages in inflammation and tissue repair, future studies incorporating detailed phenotypic and functional analyses will be required to clarify the contributions of distinct macrophage subsets to PG pathogenesis across species.

A previous study demonstrated that C5a regulates leukocyte migration and NLRP3 activation through ROS generation in a murine model of peritonitis (Khameneh et al, 2017); however, the role of C5a in PG remains poorly understood. An important finding of this study is the differential therapeutic impact of corticosteroid treatment and C5a pathway inhibition on wound healing in the brequinar-induced PG mouse model. Systemic corticosteroids have established their position as the standard treatment for PG in clinical practice owing to their potent anti-inflammatory effects (Partridge et al, 2018). However, corticosteroids exert broad immunosuppressive actions and are known to impair physiological processes essential for tissue repair, including keratinocyte proliferation, fibroblast activation, and angiogenesis (Carolina et al, 2018; Jozic et al, 2017; Wicke et al, 2000). Our RNA-sequencing analysis revealed a substantial overlap in gene expression changes between vehicle- and brequinar-treated wounds compared with that in unwounded skin, with 2416 genes commonly upregulated in both groups, suggesting that PG-like lesions share many molecular features with physiological wound healing. These findings may explain why prednisolone failed to improve wound healing even though it suppressed inflammatory gene expression and neutrophil activation in the PG mouse model. In contrast, selective inhibition of C5a signaling appears to attenuate pathogenic neutrophil-induced inflammation while preserving essential wound-healing processes. Treatment with avacopan was associated with reduced neutrophil-associated inflammation and improved wound healing. However, the precise mechanisms underlying these effects remain to be determined.

Furthermore, avacopan has been clinically applied for the treatment of anti-neutrophil cytoplasmic antibody–associated vasculitis, a condition characterized by excessive neutrophil activation and NET formation, which are key drivers of vascular inflammation (Shiratori-Aso and Nakazawa, 2023). In a mouse model of anti-neutrophil cytoplasmic antibody–associated necrotizing and crescentic glomerulonephritis, oral avacopan was evaluated to determine its effects on disease severity and complement inhibition in a dose-dependent manner; administration of 4 mg/kg twice daily significantly reduced disease severity (Xiao et al, 2014). On the basis of these dose–response data and the demonstrated efficacy of the 4 mg/kg twice-daily regimen, this study employed an 8 mg/kg once-daily dosing schedule as a pharmacologically equivalent regimen. In murine models, it exhibited greater efficacy than corticosteroids in promoting wound healing, indicating its potential suitability for future clinical application in PG.

This study has several limitations. First, transcriptomic analyses were performed using bulk RNA sequencing, which precludes precise identification of cell-type–specific cytokine sources. Immunofluorescence staining demonstrated prominent expression of NLRP3, caspase-1, and IL-1β in Ly6G-positive neutrophils, suggesting that neutrophils may be a major source of inflammasome activation in this model. However, the contribution of other cell types, including macrophages, keratinocytes, and endothelial cells, cannot be excluded and warrants further investigation. Moreover, the interplay among C5a signaling, oxidative stress responses, and NLRP3 inflammasome activation is inferred primarily from transcriptomic and pharmacologic data, and direct mechanistic evidence is lacking. Second, a direct comparison between PG lesions and normally healing wounds in human samples was not feasible. As a result, some of the inflammatory and transcriptomic changes observed in PG lesions may overlap with physiological wound-healing processes rather than being entirely disease specific. Third, the temporal dynamics of immune cell infiltration were not assessed. Future studies employing single-cell or spatial transcriptomics and longitudinal sampling will be essential to refine our understanding of PG pathogenesis. Fourth, given the fundamental differences between human and murine wound-healing mechanisms, with wound contraction playing a dominant role in mice, the absence of experimental suppression of wound contraction represents a limitation of this study. Further evaluation will be required to more precisely assess the effects of corticosteroids and avacopan on wound healing in this PG mouse model. Fifth, female mice are commonly used in cutaneous wound-healing and inflammatory skin models; however, sex-based differences were not assessed and warrant investigation in future studies. Finally, enrofloxacin was selected because it is a widely used and clinically established antimicrobial agent that can be conveniently administered through drinking water. However, we did not evaluate other classes of antibiotics in parallel. In addition, antibiotic treatment itself may have influenced local inflammatory responses and wound-healing processes through alterations in the skin microbiota and host immune responses.

**Figure 12:**
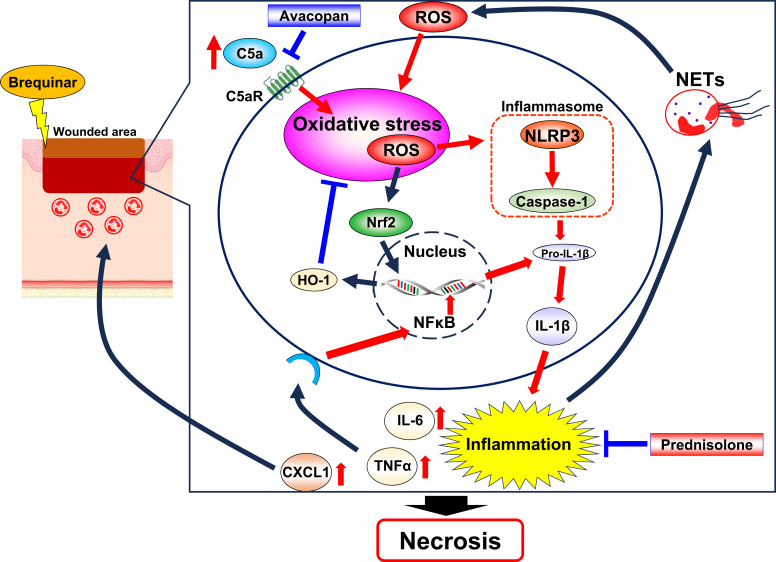
**Schematic model summarizing the pathogenic mechanisms in the brequinar-induced murine model of PG.** We propose that topical application of brequinar induces the activation of C5a signals, which may contribute to oxidative stress and NLRP3/caspase-1 inflammasome activation. This cascade results in increased production of proinflammatory cytokines, enhanced neutrophil activation with NET formation, and activation of Nrf2 signaling. Systemic administration of prednisolone and avacopan suppresses inflammatory cytokine expression and NET formation. NET, neutrophil extracellular trap; PG, pyoderma gangrenosum.

In conclusion, our study establishes a modified brequinar-induced PG-like mouse model that recapitulates key immunopathological features of human PG and identifies the activation of the NLRP3/caspase-1 inflammasome, which is associated with type 1 inflammation and NET formation (Figure 12). These findings provide a mechanistic framework for PG pathogenesis and support the development of targeted therapies aimed at innate immune dysregulation in this challenging disease.

## Materials and Methods

### Human biopsy samples

Skin biopsy specimens were obtained from patients diagnosed with PG on the basis of established clinical and histopathological criteria (Maverakis et al, 2018). Normal human skin samples were obtained from surplus, noninflamed skin tissue of non-PG patients undergoing elective dermatologic surgery and were used as controls. All procedures involving human samples were approved, and written informed consent was obtained from all participants in accordance with the Declaration of Helsinki. Written informed consent for the publication of clinical images was also obtained from all participants.

### Mice

All experiments were approved and carried out in accordance with the approved guidelines. Female C57BL/6 wild-type mice (aged 8 weeks) were purchased from SLC (Shizuoka, Japan). OKD-Luc (Keap1-dependent oxidative stress detector, No.48: B6-ARE-NRF2/LUC) mice were kindly provided. Female mice aged 8 weeks were used for all experiments. Mice were bred and maintained under specific pathogen-free conditions. For genotyping, genomic DNA was isolated from mouse tails using the Wizard Genomic DNA Purification Kit (Promega), following the manufacturer’s instructions.

### Development of brequinar-induced modified PG mouse model

Full-thickness excisional wounds were created as previously described (Uchiyama et al, 2014). Briefly, mice were anesthetized, and dorsal hair was removed by topical application of cream 24 hours before wounding, and 5-mm full-thickness excisional dorsal skin wounds were created using a sterile disposable biopsy punch (KAI MEDICAL, Gifu, Japan). Brequinar was purchased from Tokyo Chemical Industry (Tokyo, Japan). Brequinar was diluted in DMSO at 37 °C and mixed with petroleum jelly (Vaseline). For induction of PG-like lesions, 20 mg of 0.7% brequinar ointment or the same amount of DMSO-containing Vaseline was topically applied to the wound site every day for 9 consecutive days as a vehicle control. To prevent secondary bacterial infection associated with topical brequinar application, mice received antibiotics (enrofloxacin: 0.17 mg/ml, Kyoritsu Seiyaku, Tokyo, Japan) in their drinking water throughout the experimental period. For therapeutic intervention studies, prednisolone (10 mg/kg/day) or avacopan (8 mg/kg/day) diluted in 0.5% w/v methyl cellulose 400 solution (FUJIFILM Wako Pure Chemical, Osaka, Japan) was administered orally once daily for 9 consecutive days after wound creation and brequinar application. The same amount of methyl cellulose was administered as a vehicle control treatment. Wound areas were digitally photographed at the indicated time points, and wound size was measured using ImageJ software (version 1.48, National Institutes of Health, Bethesda, MD).

### Histological examination and immunofluorescence staining

Human skin biopsy was fixed in 20% formalin and embedded in paraffin. Murine skin was removed, fixed in formalin, and embedded in paraffin. A total of 4-μm sections were stained with H&E. Re-epithelialization was quantified using H&E-stained sections by measuring the epithelial tongue length extending from the wound edge toward the wound center. Measurements were performed on images captured by light microscopy and analyzed using ImageJ software. For immunohistochemical staining with paraffin sections, deparaffinized sections were boiled for 10 minutes for antigen retrieval. Sections were treated with endogenous peroxidase-blocking reagent (Dako, Agilent) for 5 minutes and protein block (Dako) for 10 minutes at room temperature. The sections were then incubated with the indicated primary antibodies overnight at 4 °C, followed by Alexa 488– and 568–conjugated secondary antibodies. Sections were counterstained with DAPI to visualize nuclei and mounted in ProLong Gold antifade reagent (Life Technologies, Carlsbad, CA). Images were photographed using Andor BC43 CF (Oxford Instruments, Abingdon-on-Thames, United Kingdom).

### Antibodies

Antibodies, their dilutions, and their sources were as follows: anti-MPO antibody (1:100, RB-373-A0, Thermo Fisher Scientific), anti-CD68 antibody (used undiluted, 413791, Nichirei Biosciences, Tokyo, Japan), anti-ELANE antibody (1:100, HPA066836, Atlas Antibodies, Stockholm, Sweden), anti-Histone H2B antibody (1:1000, ab134211, Abcam), Hoechst 33342 (1:250, 62249, Thermo Fisher Scientific), anti–heme oxygenase-1 antibody (1:200, ab13243, Abcam), anti-NLRP3 antibody (1:111.4, ab263899, Abcam), anti–caspase 1 (cleaved Asp210) antibody (1:100, PA5-38099, Thermo Fisher Scientific), anti–IL-1β (cleaved Asp116) antibody (1:100, PA5-105048, Thermo Fisher Scientific), antimouse IL-6 antibody (1:40, AF-406, R&D Systems), antihuman IL-6 antibody (1:166.7, MAB-2061, R&D Systems), and anti-Ly6G antibody (FITC conjugate, 1:200, #88876, Cell Signaling Technology, Danvers, MA). Alexa 488– and Alexa 568–conjugated secondary antibodies were obtained from Invitrogen (1:250, Carlsbad, CA).

### Real-time reverse transcriptase PCR

Total RNA from skin tissue and cells was isolated using a RNeasy Mini Kit (Qiagen, Valencia, CA) and was subjected to reverse transcription using a GoScript Reverse Transcription System for RT-PCR (Promega) according to the manufacturer's instructions. qRT-PCR was performed with the SYBR system (Applied Biosystems, Foster City, CA) using QuantStudio3 real-time PCR instrumentation (Applied Biosystems) according to the manufacturer's instructions. SYBR probes and primers for *TNFα*, *IL1β*, *IL6*, *CXCL1*, *CCL2*, *S100A8, S100A9*, *HMOX1*, and *18S* were purchased from Sigma-Aldrich (St. Louis, MO) and Takara Bio (Otsu, Japan) and Integrated DNA Technologies (Coralville, OH). As an internal control, the levels of 18S mRNA were quantified in parallel with the target genes. Normalization and fold changes were calculated using the comparative Ct method.

### RNA-sequencing data analysis

Total RNA from skin tissue was used, and mRNA expression profiling was performed in the Core Facility Management and Technical Collaboration Center at Gunma University. Library preparation was carried out using the KAPA mRNA HyperPrep kit (KAPA BIOSYSTEMS, KK8580), according to the manufacturer’s instructions. Library quality was assessed using an Agilent 4200 TapeStation (Agilent). Paired-end sequencing was performed on a NextSeq 2000 platform (Illumina). Reads were aligned to the mm10 assembly of the mouse genome (National Center for Biotechnology Information Build 38, obtained from the UCSC Genome Browser website) using the STAR aligner. Gene-level raw count data were used for downstream analyses. RNA-sequencing data were analyzed using the iDEP Genomics Suite (https://bioinformatics.sdstate.edu/idep/). Relative log expression plots were used to assess data normalization and quality. Differential gene expression analysis was performed using the Wald test for pairwise comparisons. For human samples, publicly available RNA-sequencing datasets comparing PG lesional skin with normal skin were analyzed. In mouse samples, brequinar-treated skin was compared with vehicle-treated skin and with unwounded skin. Gene Ontology terms (*P* < .01, fold change > 4 for human and *P* < .05, fold change > 1.5 for mouse) were obtained using ToppGene (https://toppgene.cchmc.org/). Ingenuity Pathway Analysis (Ingenuity Systems, www.ingenuity.com) was used for the identification of diseases and functions terms, upstream regulator analysis, and network analysis. *P*-values were adjusted for multiple testing where applicable. Heat maps depict gene-wise scaled expression values on the basis of log-transformed counts. For transcriptomic analysis, publicly available human RNA-sequencing data from lesional skin of patients with PG (n = 8) and healthy control skin (n = 8) were reanalyzed from the Sequence Read Archive (BioProject accession PRJNA590986) and, together with the newly generated mouse RNA-sequencing data from this study, were deposited in the Gene Expression Omnibus under accession number GSE337765 (https://www.ncbi.nlm.nih.gov/geo/query/acc.cgi?acc=GSE337765).

### Statistical analysis and sample numbers

*P*-values were calculated using Student's *t*-test (2 sided) or by 1-way ANOVA followed by the Tukey-Kramer test. Data analysis was performed with R, version 4.3.1 (The R Foundation for Statistical Computing: https://www.r-project.org/). Error bars represent SEM, and the numbers of experiments (n) are indicated.

## Ethics Statement

### Human biopsy samples

All procedures involving human samples were approved by the Ethics Committee of Gunma University Graduate School of Medicine (#HS2025-238), and written informed consent was obtained from all participants in accordance with the Declaration of Helsinki. Written informed consent for publication of the clinical images was also obtained from all participants.

### Mice

All experiments were approved by the Gunma University Animal Care and Experimentation Committee (#24-020) and carried out in accordance with the approved guidelines. Mice were bred and maintained in the Institute of Experimental Animal Research of Gunma University under specific pathogen-free conditions.

## Data Availability Statement

Human transcriptomic data were obtained from the Sequence Read Archive (BioProject accession PRJNA590986; https://www.ncbi.nlm.nih.gov/sra/PRJNA590986). Raw and processed mouse RNA-sequencing data have been deposited in the Gene Expression Omnibus under accession number GSE337765 and are publicly available at https://www.ncbi.nlm.nih.gov/geo/query/acc.cgi?acc=GSE337765. Other data supporting the findings of this study are available from the corresponding author upon reasonable request.

## ORCIDs

Takeshi Araki: http://orcid.org/0000-0002-6778-6816

Akihiko Uchiyama: http://orcid.org/0000-0002-2169-2427

Rimu Takata: http://orcid.org/0009-0007-5637-2224

Keiji Kosaka: http://orcid.org/0000-0001-5112-276X

Mayu Ohtaka: http://orcid.org/0009-0002-2416-3333

Shintaro Saito: http://orcid.org/0000-0001-5736-8066

Takayuki Shuto: http://orcid.org/0009-0004-1255-688X

Akiko Sekiguchi: http://orcid.org/0000-0002-7016-337X

Sachiko Ogino: http://orcid.org/0000-0002-5026-7972

Yoko Yokoyama: http://orcid.org/0000-0002-9878-7555

Ryoko Torii: http://orcid.org/0000-0002-1083-9550

Yuki Watanuki: http://orcid.org/0009-0006-1263-8679

Sei-ichiro Motegi: http://orcid.org/0000-0001-8286-0669

## Conflict of Interest

The authors state no conflict of interest.
